# Atrophie blanche complicated with lower limb infection and maggot growth: A case report

**DOI:** 10.3389/fsurg.2022.941568

**Published:** 2022-11-07

**Authors:** Bo Zhou, Haotian Qin, Yicong Huang, Quanzhen Wang, Jian Zhang, Yingfeng Xiao, Yanbin Peng, Fei Yu

**Affiliations:** ^1^ Department of Hand & Microsurgery, Peking University Shenzhen Hospital, Shenzhen, China; ^2^ Department of Bone & Joint Surgery, Peking University Shenzhen Hospital, Shenzhen, China; ^3^ National & Local Joint Engineering Research Center of Orthopaedic Biomaterials, Peking University Shenzhen Hospital, Shenzhen, China; ^4^ Department of Orthopedic Surgery, Affiliated Dongguan People's Hospital, Southern Medical University, Dongguan, China

**Keywords:** atrophie blanche, maggots, skin grafting, suppurative infection, surgery

## Abstract

**Background:**

Atrophie blanche (AB) is a thrombotic vascular disease, also known as venous vasculitis or segmental hyaline vasculitis, characterized by chronic recurring painful ulcers on the lower legs, especially the ankles. AB is a clinically rare condition, affecting 1%–5% of the population, specifically middle-aged women with an average age of 45 years, and cases of AB in children are rare. Following recovery, ivory-white atrophy spots accompanied by pigmentation and telangiectasia remain in patients. One of the complications of AB is the parasitic growth of microorganisms infecting the ischemic soft tissue undergoing necrosis in the lower limbs. Furthermore, although infection combined with microbial parasitism is a type of surgical site infection, myiasis is particularly rare, which may warrant limb amputation or may even be life-threatening. Understanding the complications of AB may help in early and timely surgical debridement as well as wound repair. Summarizing the knowledge and treatment strategies of AB and formulating clinical strategies and guidelines for AB management with insights from relevant cases are important.

**Case summary:**

A 59-year-old woman was hospitalized due to repeated ulceration of the skin of the right lower leg for 3 years, aggravation, and maggot growth for 3 days. In the previous 3 years, the skin and soft tissue of the right calf had become ischemic, necrotic, and infected, but the patient did not seek any medical treatment. Subsequently, 2 years ago, she was diagnosed with AB at the dermatology department of our hospital. After hormone treatment, her right leg improved. However, 1 year ago, the skin and soft tissue of the right leg again became ischemic, necrotic, and infected. This time, the patient did not seek medical treatment and applied musk on her wound. The wound deepened, resulting in the exposure of the tendon and some bones. In addition, a large number of maggots and microorganisms grew in and infested the wound for 3 days before the patient came to our department for treatment. Debridement of the necrotizing infected site on the right lower leg combined with negative pressure vacuum sealing drainage were performed twice within 16 days after admission. Simultaneously, antibiotics were given systemically. On the 17th day after admission, the wound appeared clean, myiasis had resolved, and the growth and coverage of the granulation tissue on the wound were satisfactory. Subsequently, debridement of the infected site on the right leg, removal of skin of the right thigh, and autologous free skin grafting were performed. After 10 days, the wound was clean, all skin grafts had survived, and wound repair was satisfactory. Finally, the patient was discharged after 38 days of hospitalization.

**Conclusion:**

Although AB is rare, leukodystrophy requires specialized treatment and regular follow-up. If lower limb infection and maggot growth occur simultaneously, self-treatment should be avoided and medical attention must be sought immediately. Early implementation of wound debridement and anti-infective treatment combined with wound repair, which should be performed after cleaning the wound, is advised.

## Introduction

Atrophie blanche (AB) is a rare disease, affecting 1%–5% of the population. It most commonly affects middle-aged women, with an average age of 45 years. However, a few cases of AB have been identified in children (1). AB causes chronic, recurring, and painful ulcers on the calves (2). There are several hypotheses on the etiology of livedoid vascular disease, but the precise underlying mechanism is not yet known. Furthermore, superficial dermal vascular obstruction occurs in AB, followed by skin ulcers (3). After wound healing, ivory-white atrophic spots may remain in patients, which are frequently accompanied by pigmentation and telangiectasia of small vessel disease, also known as livedo vasculitis or segmental hyaline vasculitis. Vascular embolism is the most common histopathological change in almost all infections (4). In clinical settings, AB is relatively rare because chronic occlusion of lower limb blood vessels can easily occur, accompanied by lower limb soft tissue ischemia and necrosis infection. However, microbial parasitic growth, such as growth of bacteria, fungi, and anaerobic bacteria, can occur in patients with AB. There is no effective strategy for treating white atrophy based on existing case reports, case series, or expert experience to achieve the therapeutic goals of reducing pain and preventing atrophic scar formation (5). At present, the treatment for AB primarily includes anticoagulants, antiplatelet drugs, glucocorticoids, thrombolytic agents, intravenous immunoglobulins, anti-inflammatory drugs, vitamins, hyperbaric oxygen, and ultraviolet phototherapy (6). In agricultural and pastoral areas and regions with poor sanitation, infected tissues (such as the eyes, ears, mouth, skin, digestive tract, and genitourinary tract) are accompanied with numerous maggots, while microbial infection and parasitism are rarely observed (7–9). With improved quality of life and advancements in medical technology, particularly aseptic surgical techniques and antibiotics, AB accompanied with lower limb infection and maggot growth has become a rare occurrence. Clinical data, diagnosis, and treatment of AB are reported in this study.

## Case presentation

### Chief complaints

A 59-year-old woman was admitted to a hospital because of repeated ulceration on the skin of the right calf for 3 years, aggravation, and maggot growth for 3 days.

### History of present illness

Three years ago, the patient showed distal skin ulceration on the lower right leg with blisters and erythematous skin. She felt severe pain during walking, which could be relieved by rest. The pain recurred, and all symptoms were progressive. However, the patient ignored these signs and did not seek any medical advice or treatment. Two years ago, the patient visited the dermatology department of our hospital for treatment and was diagnosed with AB. Her condition improved after drug treatment (compound danshen tablets, tritodoside tablets, calcium hydroxybenzene sulfate capsules, aspirin, and dipyridamole tablets—panenstin); however, she developed ulceration again on the distal anterior skin 1 year ago. Furthermore, the patient self-medicated multiple times with musk, but this topical treatment did not heal the wound and instead led to aggravation. However, the patient ignored this condition and did not seek treatment at the hospital. Several maggots were observed in the ulcerated area on the far anterior side of the skin (3 days earlier), accompanied by oozing foul-smelling, yellowish-green fluid and fever (highest temperature: 39 °C). Furthermore, she had symptoms such as nonpersistent fever, feeling cold in extremities, shortness of breath, and drenching sweats. However, she did not experience any chest tightness or fatigue. The patient visited our emergency department for blood examination, and the results were as follows: total white blood cells = 13.4 × 10^9^/L, Rt-c-reactive protein (CRP) = 123.86 mg/L, hs-CRP > 5.0 mg/L, and procalcitonin (PCT) = 0.18 ng/ml. The patient was admitted to our hospital because of right lower leg gangrene with infection. The patient showed poor mental health, sleep, and appetite since the time of this disease occurrence and low gastric feeding, minimal food intake, normal stools, and weight loss of 3 kg in approximately 1 month.

### History of past illnesses

The patient had a history of lumbar disc herniation and was allergic to cephalosporins.

### Personal and family histories

The patient had no family history of hereditary, immunological, or psychiatric diseases.

### Physical examinations

The patient was brought to the department in a wheelchair. Physical examination revealed multiple ulcers scattered over the skin of lower limbs, with ulcers on the right calf being more prominent than those on the left calf. Some ulcers were scabbed, with no varicose veins. A large ulcerated wound (approximately 15 cm × 10 cm) was observed extending from the anterior tibia to the dorsum of the right calf distal to the fascia, spreading deep within the fascial layer with nerves, blood vessels, tendons, and bony excrescence. Darkening of the soft tissue, visible crawling of several maggots, and flushing of the skin around the edges of the wound were observed. However, no subcutaneous wave sensation was observed. Bilateral femoral and popliteal artery pulsatilities were good; pulsatility of the right dorsal foot artery was weaker than that of the contralateral artery; and bilateral posterior tibial artery pulsatility was palpable. There was limited bilateral ankle movement, with 10° of dorsiflexion and 5° of extension of the toes on the right and 20° of dorsiflexion and 10° of extension of the toes on the left. Mild tenderness over the lumbar spine was observed. No pain on percussion and no sensory numbness were noted in both lower limbs.

### Laboratory examinations

The blood test results were as follows: Total number of leukocytes = 13.4 × 10^9^/L; Rt-CRP = 123.86 mg/L; hs-CRP > 5.0 mg/L; PCT = 0.18 ng/ml.

### Imaging examinations

**Figure 1:**
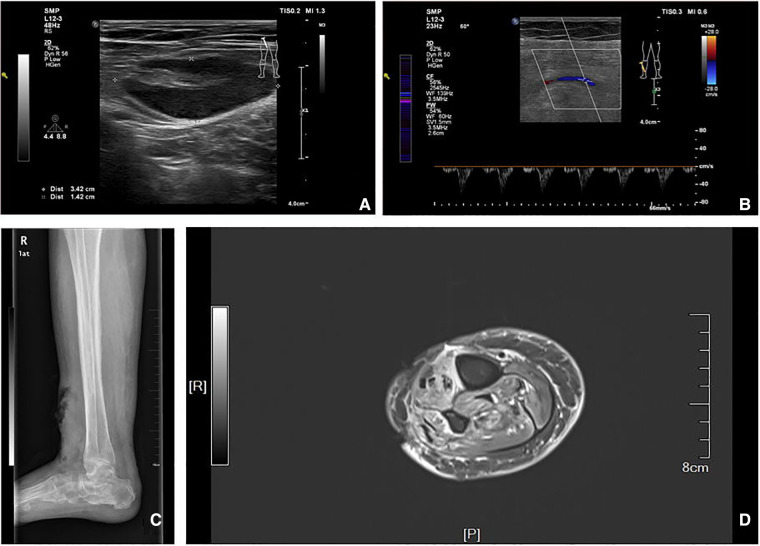
(**A,B**) Arteriovenous color ultrasound images of the lower limb. (**C**) Lateral view of the ankle. (**D**) MRI image of the limb.

Figure 1 shows the results of the physical examination of the patient. The diagnosis, treatment, and management of the disease are discussed in the following sections.

## Final diagnosis

The patient was diagnosed with the following conditions: gangrene and infection of the right lower leg with cutaneous soft tissue defects; blood vessel, nerve, tendon, and bone exteriorization; AB; chronic ulceration of both lower limbs; and lumbar disc herniation.

## Treatment

Step 1: After examination, the patient was administered AB maintenance treatment (compound danshen tablets, polyside, calcium hydroxybenzene sulfate capsule, aspirin intestinal G, and dipyridamole tablets—panshengtin).

Step 2: Wound disinfection and systemic anti-infection treatment using empirical cefuroxime sodium intravenous drip anti-infection treatment and drugs was administered.

Step 3: To eliminate surgical contraindications, such as coagulation and serious cardiopulmonary disease, right calf necrosis infection lesions debridement and ventricular septal defect negative pressure drainage (lumbar hard combined with anesthesia in the supine position tourniquet on the root of the right thigh) were performed twice within 16 days at an interval of 1 week. The discharge was collected for inspection, and superficial purulent secretions and maggots were removed during the operation. Large necrotic and purulent ulcerated wounds in front of the tibia at the distal end of the right leg and scabby ulcers on the skin of both feet combined with bilateral toe deformity were observed. The extensor hallucis longus tendons, anterior tibial tendon, and peroneal short tendon were necrotic, rotten, and liquefied. The wound was covered with maggots. A scar-like and swollen old granulated tissue was observed around the wound. Ankle joint mobility was noted to be poor. When the skin and soft tissue along the anterior tibial tendon were cut (approximately 6 cm), we observed that the maggots were alive in the upper segment of the tendon. Later, the inactivated tendon was expanded, pulled out, and cut off at a higher position. The inflammatory granulated tissue around the wound was scraped with a curette. During the operation, the adhesive tendon and ankle joint were loosened and the maggots and worm eggs were removed. The hypertrophic scar tissue was cut, especially the large scar tissue inside the ankle. Obvious edema in superficial and deep peroneal nerves was observed. The distal end of the superficial peroneal nerve was liquefied and dissolved. The dorsal foot artery was continuous, while its vascular elasticity was poor. The left unclosed wound was later connected to negative pressure on vacuum sealing drainage (VSD) material suction with a negative pressure of approximately 0.04 kPa.

The results of wound secretion culture revealed the presence of *Staphylococcus aureus* susceptible to cefuroxime sodium and levofloxacin. Based on wound secretion culture results and empirical judgment, an injection of levofloxacin was administered as systemic anti-infection treatment after pharmaceutical consultation.

**Figure 2:**
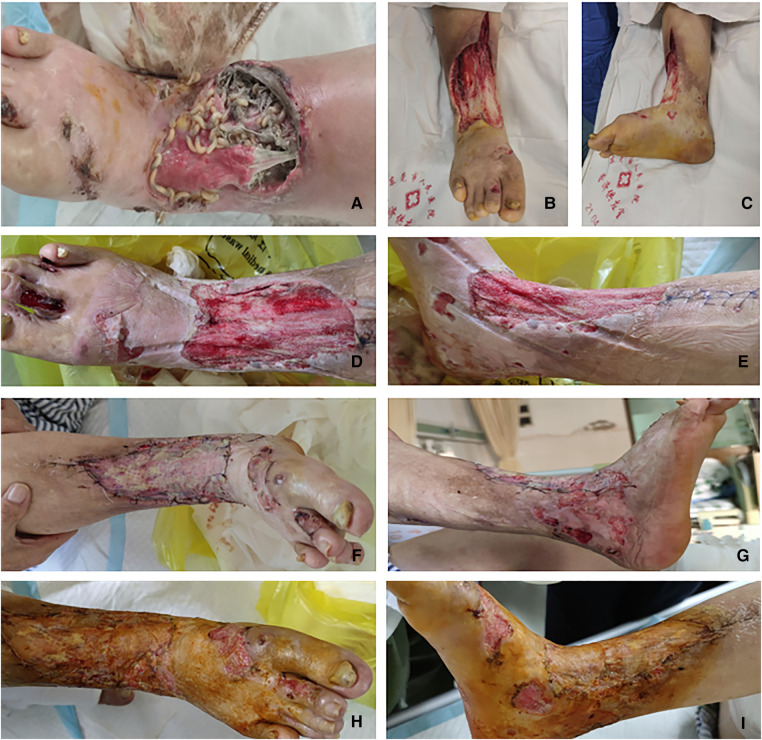
(**A**) Condition of the preoperative wound. (**B,C**) Condition after the first debridement of the wound. (**D,E**) Condition after the second debridement of the wound. (**F,G**) Condition of all skin grafts on the 10th day after skin grafting; all grafts survived. (**H,I**) The wound healed after skin grafting in half a month.

Step 4: After 17 days, the wound was clean, with no maggot growth, and wound granulation tissue growth coverage was satisfactory. Right calf infection lesion debridement expansion and right thigh skin and autologous free skin grafting were then implemented (specific operation steps are as follows). After administering spinal anesthesia, the patient was placed in a supine position. The surgical area of the right lower limb was routinely disinfected and paved. The surgical area was measured, and a thick skin blade in front of the right thigh was removed. Skin defects on the right calf and foot, inflammatory granulation tissue, and surrounding scar tissue were removed. Bleeding was stopped after repeated washing. The 8 cm × 10-cm acellular allogeneic dermal material was transplanted to cover the soft tissue defect wound, with an intermittent suture as the graft base. After trimming the autologous skin sheet to an appropriate size, it was transplanted onto the soft tissue wound on the leg and foot and packed with the front line and oil yarn-fixed skin grafting area (sterile dressing bandage hemostasis fixation). After 10 days, we observed that all skin grafts had survived and wound repair was satisfactory. The patient was discharged after 38 days of hospitalization (Figure 2).

## Outcome and follow-up

The above procedures were uneventful. The wound on the right lower leg healed after the surgery. The surrounding tissue displayed no apparent redness and exudation as well as no crawling of maggots. The skin graft survived after discharge. We also informed the patient to pay attention to the sanitation and cleanliness of the area surrounding the wound and suggested to take regular treatment for AB.

Regular follow-up visits to the outpatient clinic 2 and 4 months after discharge revealed old scar hyperplasia and complete wound healing. However, to the best of our knowledge, patients with AB might suffer from recurrent lower limb ulcers during the follow-up period.

## Discussion

AB, also known as livedo vasculitis or hyaline segmental vasculitis, is a chronic, relapsing, rare skin disease that affects the distal lower extremities (10–12). It is a relapsing vaso-occlusive disease that occurs in the lower legs, ankles, and back of the foot and is characterized by erythema, purpura, and painful ulcers. Furthermore, AB leaves a characteristic ivory-white scar, telangiectasia, and pigmentation after healing (3). The initial onset of AB is characterized by the symmetrical appearance of erythema, purpura, and macules on the dorsa of lower legs, ankles, and feet, partly nodular and vesicular occurrence. This is followed by the development of sharp pain, ulcers, oozing fluid, and crusting on the disease base, which is recurrent, difficult to heal, and severe during winter. In addition, the long-term recurrence leaves ivory-white or yellow-white atrophic scars, which are stellate, punctate, or irregular in shape, accompanied by telangiectasia and pigmentation (13), leading to delayed wound healing, microbial infection, and further development into edema and thrombosis (14). It is necessary to distinguish AB from allergic vasculitis, cutaneous nodular polyarteritis, and allergic purpura. Although AB is treated differently, there is no specific treatment for it. Moreover, delayed or improper disinfection treatment can lead to secondary infection or even amputation. Therefore, timely surgical treatments are required, and the wound needs to undergo skin grafting or flap repair.

The patient in this study had leukodystrophy but did not seek medical advice when secondary right lower leg ischemia combined with skin soft tissue infection and necrosis occurred. She treated herself by externally applying musk on the wound, which aggravated the lower right leg infection, resulting in skin soft tissue defects as well as microbial infection, myiasis, and parasitic infestation. Hence, timely medical treatment can avoid wound myiasis.

Myiasis typically occurs in individuals working in pastoral areas and in field workers in tropical and subtropical regions; however, clinical cases of maggot infestation are relatively rare. Several cases of myiasis caused by Diptera have been reported in Iran (15). Rowicki and Pietniczka-Załęska (13) reported the first incident of filiform maggot infection in a patient with cervical lymph node metastasis. In addition, Sangmala et al. (14) reported a case of a 3-month-old patient with cutaneous psoriasis caused by big-head golden flies. This fly (*Chrysomya megacephala*) is more commonly referred to as “oriental blue fly.” Sunny et al. (16) reported a case of a 60-year-old male patient with leg ulcers and maggot larvae. A 3-year-old boy from a rural area developed myiasis in the mouth cavity with severe gingivitis after being infected by *Oestrus ovis* (Sheep nasal bot fly) (15). Similarly, a 74-year-old woman in Gonabad was reported to have nasal myiasis caused by *Chrysomya bezziana* (Old World screwworm fly) (17). Once confirmed, nasal myiasis should be treated timely and appropriately to prevent further tissue damage and reduce complications (18).

However, no cases of AB lower extremity infection complicated with maggot parasitism and treatment experience have been reported. The specificities of AB are as follows: (1) patients with AB suffer from repeated spontaneous ulceration on the lower legs, develop difficult-to-heal wounds, and become susceptible to microbial parasitic infections (e.g., infections caused by flies and maggots); (2) such patients have suboptimal self-healing ability of the skin, which may lead to secondary aggravation of infection and thereby large areas of tissue necrosis and defect formation. These patients occur concomitant dissection of the middle and lower segments of the right lower leg with suboptimal blood supply circulation, thin local skin conditions, such that tissue infection and necrosis occur; and (3) lack of adequate attention, improper treatment of wounds, and lack of the usage of sterile bandages can result in parasitic multiplication and growth of maggots, thereby leading to an increase in the amount of excrement, which in turn creates conditions for the multiplication of maggots.

With economic development and advancement in medical science and technology, the quality of life and awareness regarding medical advances have improved. Early debridement is an effective treatment strategy for the majority of infected and necrotic wounds. A critical control measure for this disease is its prevention by improving environmental sanitation conditions of the residents, actively killing flies, and developing good personal hygiene habits. Since infection and parasitic maggots are rare in clinical practice and limited clinical data are available, there is less experience and more challenges in treating such cases. Therefore, we combined our case to summarize our opinion on when limb infection necrosis should be diagnosed, at the same time combined with AB. Necrotic tissues and maggots need to be removed as early as possible. Anti-infection treatment should be administered systemically until no maggot parasitism remains. Later, a skin graft can be administered to further repair the wound. The patient reported herein was discharged 38 days after hospitalization. The treatment course in this patient was smooth, and the expected result was achieved.

## Conclusion

This is our first case of AB complicated with lower limb infection and maggot infection. Such patients should avoid self-management and seek medical advice as early as possible. Physicians are advised to perform systemic professional treatment, while treating AB, implement wound debridement, initiate anti-infection treatment at an early stage, and perform wound repair after the wounds are clean.

## Data Availability

The raw data supporting the conclusions of this article will be made available by the authors, without undue reservation.

## Authors’ note

The authors have read the CARE Checklist (2016), and the manuscript was prepared and revised according to the CARE Checklist (2016).
